# Development of a multiplex Endopep-MS assay for simultaneous detection of botulinum toxins A, B and E

**DOI:** 10.1038/s41598-017-14911-x

**Published:** 2017-11-01

**Authors:** Osnat Rosen, Liron Feldberg, Tamar Shamai Yamin, Eyal Dor, Ada Barnea, Avi Weissberg, Ran Zichel

**Affiliations:** 1 Department of Biotechnology, Israel Institute for Biological Research, 7410001 Ness Ziona, Israel; 2 Department of Analytical Chemistry, Israel Institute for Biological Research, 7410001 Ness Ziona, Israel

## Abstract

Botulinum neurotoxins (BoNTs) are bacterial proteins that cause botulism, a life-threatening disease. The Endopep-MS assay permits rapid detection and serotypic differential diagnosis of BoNTs. The serotype-specific nature of this assay requires that each serum sample be aliquoted and individually tested, which in addition to the limited volume of clinical samples, especially in infants, points to the need for a multiplex assay. However, previous attempts to develop such an assay have been challenging, mainly due to inhibition of BoNT/A activity by the BoNT/E peptide substrate. BoNT/A and BoNT/E share the same native target protein as their substrate. We hypothesized that the steric interference between the BoNT/A and BoNT/E substrate peptides is responsible for the difficulty in simultaneously assaying these two toxins. To explore the basis for steric interference, we used the reported structure of BoNT/A in complex with SNAP-25 and modelled the structure of BoNT/E with SNAP-25. Following this thorough structural analysis, we designed a new peptide substrate for BoNT/A that maintained the assay sensitivity and allowed, for the first time, simultaneous detection of the three most abundant human botulinum serotypes. Adopting the multiplex assay will minimize the required sample volume and assay time for botulinum detection while maintaining the superior Endopep-MS assay performance.

## Introduction

Botulinum neurotoxins (BoNTs) are bacterial proteins that cause the life-threatening disease botulism and are designated as CDC category A agents^1^. Seven antigenically distinct serotypes (designated A to G) are produced by several anaerobic species: *Clostridium botulinum, Clostridium butyricum, Clostridium baratii* and *Clostridium argentinense*. BoNTs A, B, E, and rarely, F serotypes are primarily related to human illness^2^. The neurotoxin is composed of a 150-kDa protein consisting of a 100-kDa heavy chain (HC) joined to a 50-kDa light chain (LC) via a disulphide bond. All BoNT serotypes exert similar mechanisms of action on their target nerve cells: initial binding of the C-terminal portion of the HC through ganglioside and protein receptors on the presynaptic cell surface, followed by internalization and translocation within the nerve ending, mediated by the N-terminal portion of the HC^2^. Inside the nerve terminus, the LC of the toxin, a zinc-dependent endo-peptidase, cleaves the soluble N-ethylmaleimide-sensitive factor attachment protein receptor (SNARE), which involved in the fusion and release of acetylcholine. As a result, acetylcholine transmission across neuromuscular junctions is blocked and symmetric descending flaccid paralysis occurs. Each BoNT serotype cleaves a specific peptide bond on one of the three SNARE proteins. Serotype A and E cleave the 25-kDa synaptosomal-associated protein (SNAP-25) at sites that are 17 amino acids apart (SNAP-25, Q^197^-R^198^ and R^180^-I^181^, respectively). Serotypes B, D, F and G cleave vesicle-associated membrane protein (VAMP or synaptobrevin). Serotype C acts on both SNAP-25 and syntaxin.

In the United States, most cases of botulism occur in infants and result from the germination and outgrowth of ingested spores of BoNT-producing clostridia. Bacteria can colonize the large intestine of infants younger than one year of age. In 2014, 128 of the 161 (80%) confirmed cases of botulism in the U.S. were infant botulism. The age of the infants affected by this disease was between 2 and 54 weeks, with a median age of 17 weeks^3^. Laboratory diagnosis of botulism relies on detecting the toxin in serum and stool and/or bacteria in stool. However, in infants, this diagnosis is extremely challenging due to the limited amount of blood that can be drawn safely and amount required for other laboratory tests. Moreover, the amount of toxin required to induce infant botulism is obviously lower than in adults, making detection even more challenging.

Currently, the only approved therapy for botulism consists of post-intoxication antibody treatment with an antitoxin. The preparation of choice for adults is an equine-derived antitoxin, whereas for infants, a human-derived preparation, BIG-IV, is preferred due to its reduced potential side effects. In severe cases, mechanical ventilation is needed. Antitoxin cannot follow the toxin into neurons, and therefore, the clinical benefit of antitoxin administration is thought to be due to the elimination of circulating toxin, which reduces duration and/or severity of the disease. Thus, to be effective, antitoxin must be administered early in the course of intoxication. Therefore, early diagnosis of botulism by means of rapid and sensitive detection of BoNT proteins in clinical specimens is essential for successful treatment.

The mouse bioassay is the only accepted standard method for BoNT detection^4^. In this assay, a clinical sample is injected into mice in the presence or in the absence of serotype-specific antitoxin and mortality is monitored for 96 hours. However, this assay is time consuming, labour intensive, costly, and necessitates a large number of laboratory animals per each sample. To meet this challenge, endo-peptidase mass-spectrometry (Endopep-MS) *in-vitro* activity assay was developed^5^. This assay is based on the specific endo-peptidase activity of each neurotoxin serotype. The biological activity of the neurotoxin itself is used to amplify the assay signal. Briefly, BoNT is extracted from the clinical sample using serotype-specific antibodies and then incubated with a selective peptide substrate derived from the relevant BoNT native protein target. The accumulation of BoNT cleavage products is then measured by mass-spectrometry. This assay has been shown to be suitable for the detection of all botulinum serotypes in numerous matrices, such as serum, stool, food, milk and others^6–9^. The assay only requires a few hours to complete, is highly specific and is more sensitive than the gold standard *in-vivo* mouse bioassay. Recently, we demonstrated the utility of Endopep-MS in diagnosing infant botulism in a clinically relevant timeframe^10^. The detection of toxin A was reported to clinicians in an intervention-relevant timeframe of 24 hours, days before results could be obtained from the mouse bioassay, which in any case failed to detect the low levels of toxin.

In the Endopep-MS assay, each BoNT serotype is detected separately using its own serotype-specific antibodies and a selective peptide substrate. Thus, a suspicious serum sample must be aliquoted for each individual BoNT serotype test. This in turn reduces the sample volume available for each test. Because the assay sensitivity is directly correlated with the amount of BoNT, a limited serum sample might lead to a false negative result. Therefore, attempts have been made to develop an Endopep-MS assay for the simultaneous detection of human related BoNT serotypes. Recently, Wang *et al*. examined the feasibility of developing such a multiplex Endopep-MS assay for BoNTs A, B, E and F^11^. The authors observed 90% reduced BoNT/A activity in the multiplexed format compared to the single substrate system and demonstrated that this reduced activity was the result of specific inhibition caused by BoNT/E substrate. By contrast, detection of other BoNT types was not reduced by the presence of other peptide substrates in the mixture. Therefore, the assay was set up separately for BoNT/A and for BoNT/B, E and F in a combined format.

In the present study, we report the development of a multiplex Endopep-MS assay for the simultaneous detection of botulinum A, B and E. We hypothesized that the reduced BoNT/A activity observed in the literature is related to overlap between BoNT/A and BoNT/E substrates. Based on structural analysis, a new peptide substrate for BoNT/A that does not overlap with BoNT/E was designed and was found to allow for efficient multiplex detection with similar sensitivity as the single assay.

## Results and Discussion

In a previous attempt to develop a one-step multiplex Endopep-MS assay, BoNT/A activity was shown to be specifically inhibited by the peptide substrate of BoNT/E. This specific inhibition imposed a requirement for a two-step assay in which BoNT/A and BoNT/E are assayed separately. Although in that study the BoNT/E substrate interfered with BoNT/A activity, the BoNT/A substrate did not interfere with BoNT/E activity^11^. To understand this asymmetric interference, the 3D interfaces of BoNT/A and BoNT/E substrates within their catalytic sites were examined.

### 3D interfaces of BoNT/A and BoNT/E substrates within the BoNT/A catalytic domain

Both BoNT/A and BoNT/E cleave SNAP-25. BoNT/A cleaves between residues Q^197^ and R^198^, whereas BoNT/E cleaves between residues R^180^ and I^181^. Hence, there are only 17 amino acid residues between the BoNT/A and BoNT/E cleavage sites. Each peptide substrate in the Endopep-MS assay is a SNAP-25 fragment that includes all of the amino acids that are necessary for BoNT binding and endo-peptidase activity. The sequences of the BoNT/A and BoNT/E peptide substrates described in the literature are 22 and 29 amino acids long, respectively^11^. These peptides are derivatives of amino acids 185–206 (BoNT/A) and 162–190 (BoNT/E) from SNAP-25. Hence, residues 185–190 are found in both peptides. We hypothesized that a steric overlap between these two peptide substrates is the basis for the observed binding interference, which could impair binding and hence reduce activity. Although, the same interference could theoretically affect the binding of both BoNT/A and BoNT/E substrates to their catalytic domains, this type of interference was only observed for BoNT/A.

**Figure 1:**
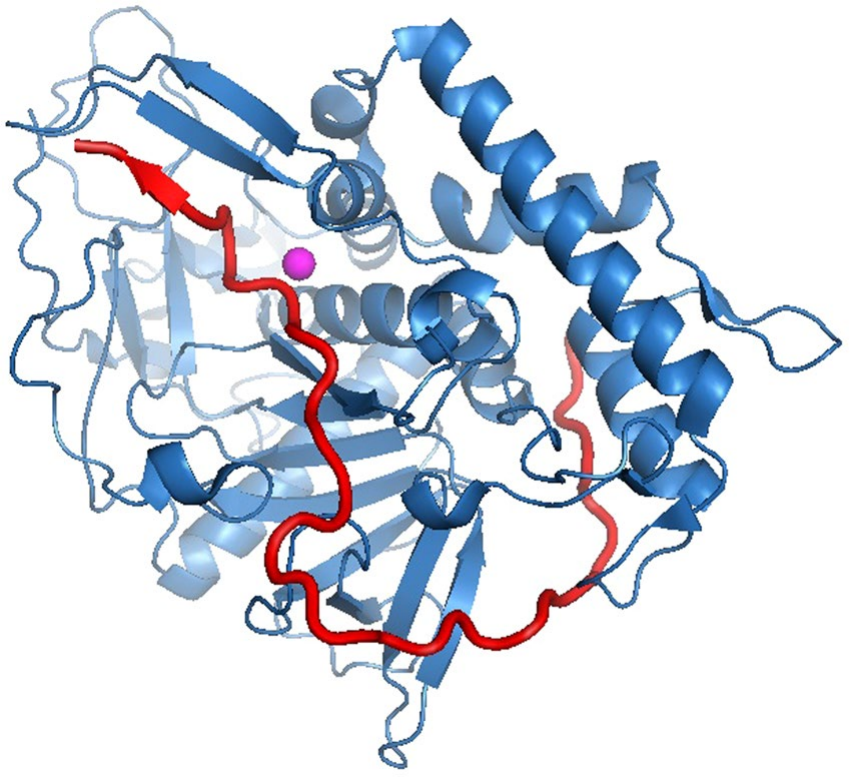
The 3D-structure of the SNAP-25 and BoNT/A catalytic domain (according to ref.^12^). The BoNT/A catalytic domain is shown in blue, and the SNAP-25 fragment 162–204 is shown in red. The catalytic zinc atom is shown in magenta.

To assess the possible interference of BoNT/E substrate with the binding of the BoNT/A substrate to the catalytic domain, the 3D-interface of both substrates with the BoNT/A catalytic domain was examined. For that purpose, the structure of SNAP-25 with the BoNT/A catalytic domain was used^12^. Since the BoNT/A substrate includes residues 185–206 and that of BoNT/E includes residues 162–190 of SNAP-25, we focused on the 3D-structure of the relevant fragment within SNAP-25 (Fig. 1, based on accession number 1XGT). An extensive network of contacts is used for SNAP-25 recognition. The substrate wraps around the BoNT/A catalytic domain and interacts broadly with it.

**Figure 2:**
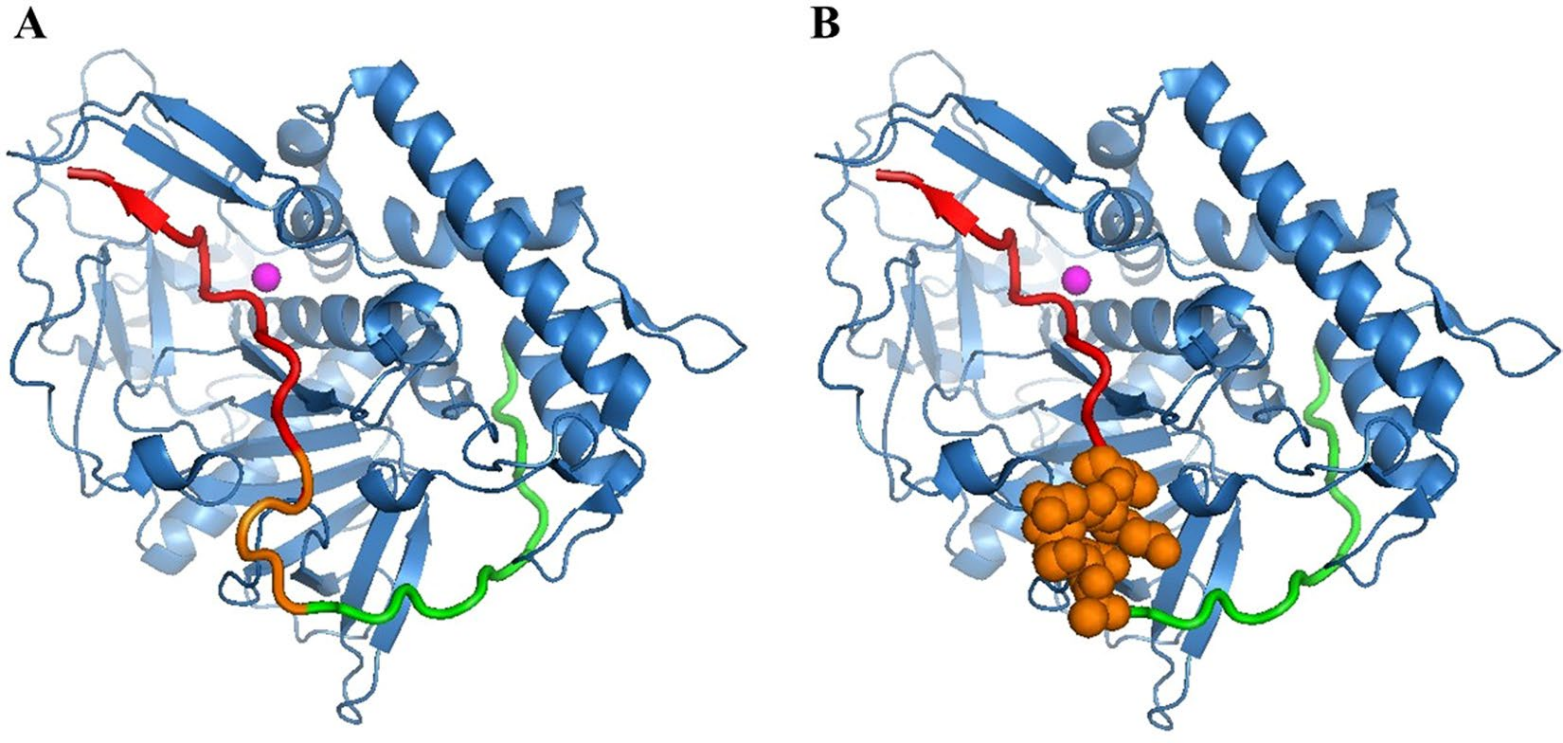
The 3D-structure of the BoNT/A and BoNT/E peptide substrates with the BoNT/A catalytic domain. (**A**) SNAP-25 fragment 167–204. The BoNT/A substrate is marked in red, BoNT/E substrate is marked in green and overlapping sequence is marked in orange. (**B**) The overlapping region of the two substrates is highlighted as orange spheres.

BoNT/A and BoNT/E substrates within the BoNT/A catalytic domain are shown in Fig. 2A (in red and green, respectively). As we assumed, there is steric overlap between these two substrates. This overlap consists of SNAP-25 position 185–190 (Fig. 2B, orange spheres). In the multiplex assay, both BoNT/A and BoNT/E substrates are added simultaneously. Therefore, both substrates can bind to the BoNT/A catalytic domain. Hence, if the BoNT/E substrate interacts with the BoNT/A catalytic domain and occupies position 185–190, it may interfere with BoNT/A substrate binding. This interference could result in the BoNT/A activity reduction reported by Wang *et al*.

### Model of the 3D-interface of SNAP-25 within the BoNT/E catalytic domain

**Figure 3:**
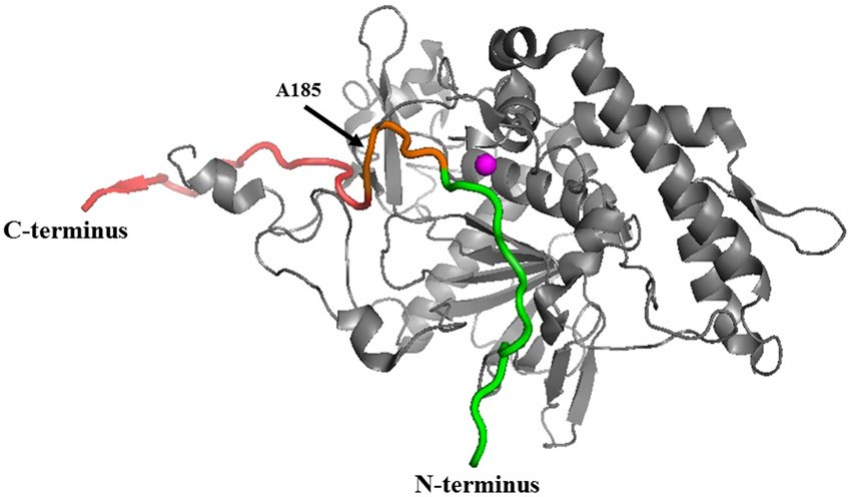
Model of the 3D-structure of SNAP-25 within the BoNT/E catalytic domain. The catalytic zinc atom is shown in magenta. The SNAP-25 fragment 167–204 is marked as the BoNT/A substrate (red) and BoNT/E substrate (green), with the overlapping sequence shown in orange. The BoNT/E catalytic domain is shown in grey.

To understand the asymmetric interference through which BoNT/A but not BoNT/E activity is impaired in the multiplex format, the structure of SNAP-25 within the BoNT/E catalytic domain was needed. However, such a structure has not been reported in the literature. Therefore, we generated a 3D model of SNAP-25-BoNT/E using the known 3D-structure of the BoNT/E catalytic domain (accession number 1T3A) and known SNAP-25-BoNT/E interactions (Fig. 3). These interactions include the hydrophobic interaction between BoNT/E Leu^166^ and SNAP-25 Ile^171^, a salt bridge between BoNT/E Arg^167^ and SNAP-25 Asp^172^ and a third interaction between the pocket of BoNT/E, which is composed of Asp^127^, Ala^128^, Ser^129^ and Ala^130^ with Asn^169^ and Gly^168^ of SNAP-25, where the side chain of Asn^169^ is docked in the pocket and Gly^168^ is located at the edge of the pocket^13^. In addition, it has been shown that removal of SNAP-25 residues 182–186 completely inhibits the ability of BoNT/E to cleave its SNAP-25 substrate, suggesting that these residues play a pivotal role in BoNT/E-SNAP-25 interactions^14^. Since those residues are not part of the active cleavage site, they may contribute to substrate binding and/or alignment by BoNT/E.

According to the model (Fig. 3), SNAP-25 residues positioned from A185 to the C-terminus do not interact with the BoNT/E catalytic domain. Therefore, the BoNT/A substrate is not expected to interfere with BoNT/E substrate binding. This may explain why BoNT/E activity was not affected in the Endopep-MS multiplex assay.

### Designing a new BoNT/A substrate for multiplexing

**Figure 4:**
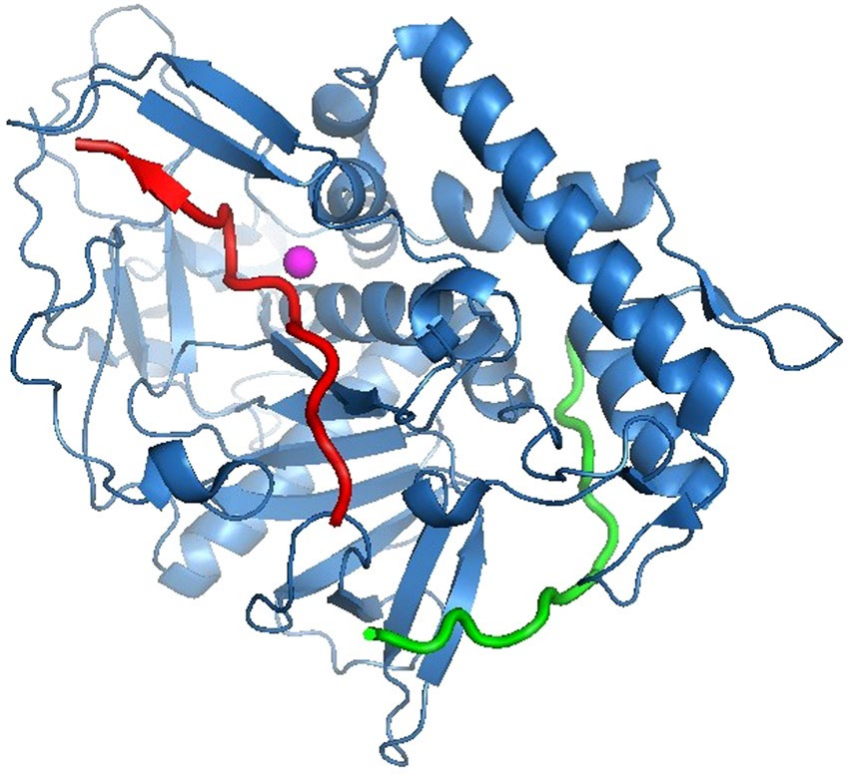
The 3D-structure of the BoNT/A catalytic domain with the newly designed BoNT/A substrate and existing BoNT/E substrate. The newly designed BoNT/A substrate (red), existing BoNT/E substrate (green), BoNT/A catalytic domain (blue) and catalytic zinc atom (magenta).

Based on our findings, we hypothesized that a new BoNT/A and/or BoNT/E substrate should be designed that would enable multiplexing without the BoNT/E substrate interfering with BoNT/A activity. For that purpose, either the C-terminus of BoNT/E, N-terminus of BoNT/A substrates, or both should be shortened. We recently found that SNAP-25 residues 167–186 comprise the optimized substrate for improved BoNT/E detection^14^. Because residues 182–186 were found to be essential for BoNT/E binding, we decided to not modify the BoNT/E substrate. In an attempt to completely avoid the steric overlap between BoNT/A and BoNT/E substrates, which is likely to cause the BoNT/A activity inhibition, we shortened the N-terminus of the BoNT/A substrate to ^189^RTRIDEGNQRATR(Nle)LG^204^. Figure 4 depicts the structure of the BoNT/A catalytic domain with the newly designed BoNT/A and existing BoNT/E substrates. It can be clearly seen that the two substrates bind separate and distinct areas of the BoNT/A catalytic domain with no overlap. This arrangement should overcome the steric interference that led to impaired BoNT/A activity and hence has the potential to be used for a multiplex Endopep-MS assay.

### Simultaneous detection of BoNT/A, B and E in a single assay

We tested the feasibility of multiplex analysis of BoNT/A, B and E using our newly designed BoNT/A substrate together with the previously reported BoNT/B and E substrates^14,15^. To this end, the three substrates for the three BoNT serotypes were mixed in a single reaction. The cleavage of serotype-specific substrates by each individual BoNT serotype was monitored. The results were compared to three individual assays, each using a single specific substrate for BoNT/A, B and E.

**Figure 5:**
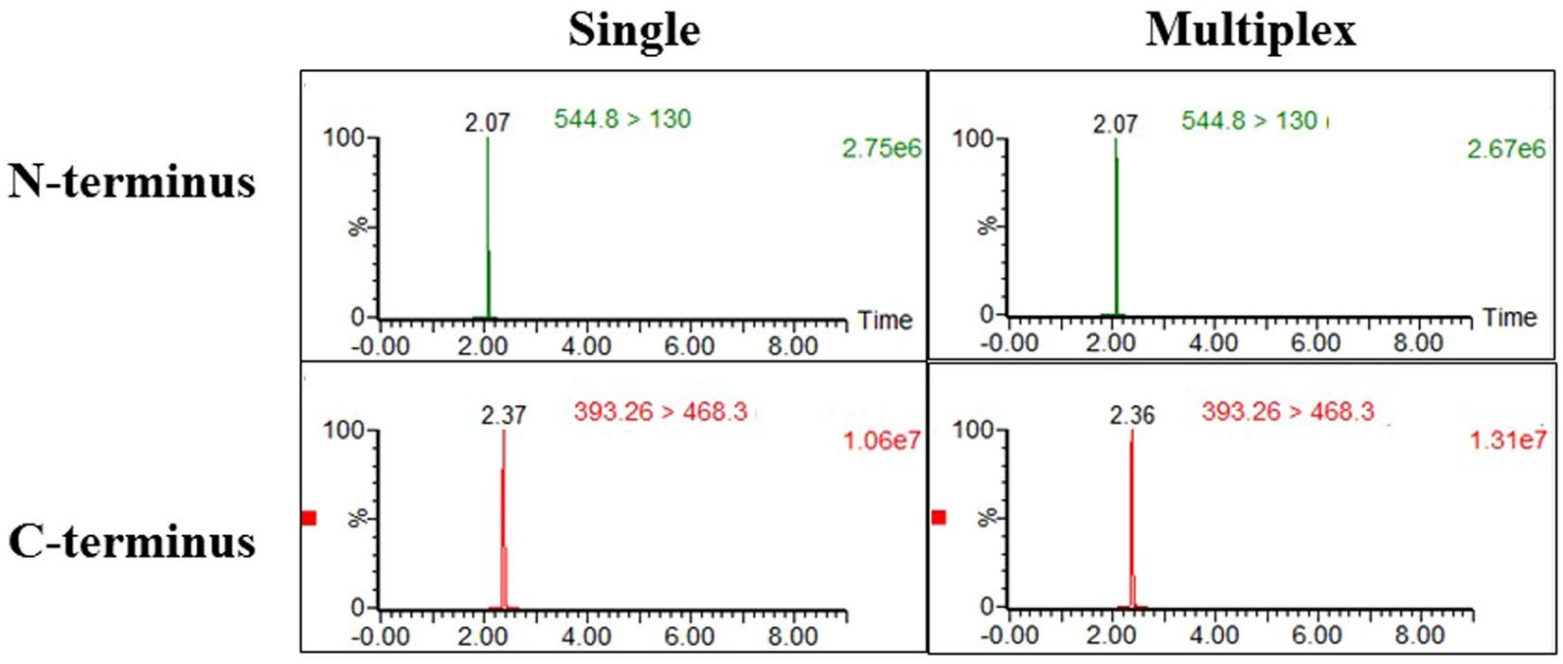
Comparing the LC-MS-MS/MRM peak intensities for N- and C-terminal cleavage products in single versus multiplex BoNT/A Endopep-MS assay. Upper panel: N-terminal cleavage product obtained in human serum (0.5 MsLD_50_/ml) from single (left) and multiplex (right) Endopep-MS assays. Lower panel: C-terminal cleavage product obtained in serum (0.5 MsLD_50_/ml) from single (left) and multiplex (right) Endopep-MS assays.

BoNT/A, B or E (0.5 MsLD_50_/ml) were spiked into human serum and extracted using magnetic beads coated with polyclonal antibodies directed to the H chain of the BoNT/A, B and E toxins. After intensive washing of the toxin-coated beads, the extracts were incubated for 5 hours at 37 °C with all three peptide substrates. Using the shortened substrate, the BoNT/A activity measured in the multiplex setup was at least as high as that in the individual assay format. As seen in Fig. 5, equal LC-MS-MS/MRM peak intensities for the N and C-terminal cleavage products were obtained in both single and multiplex Endopep-MS assays. Background MRM signal intensities were found to be similar (data not shown), leading to comparable signal to noise ratio in both formats.

**Figure 6:**
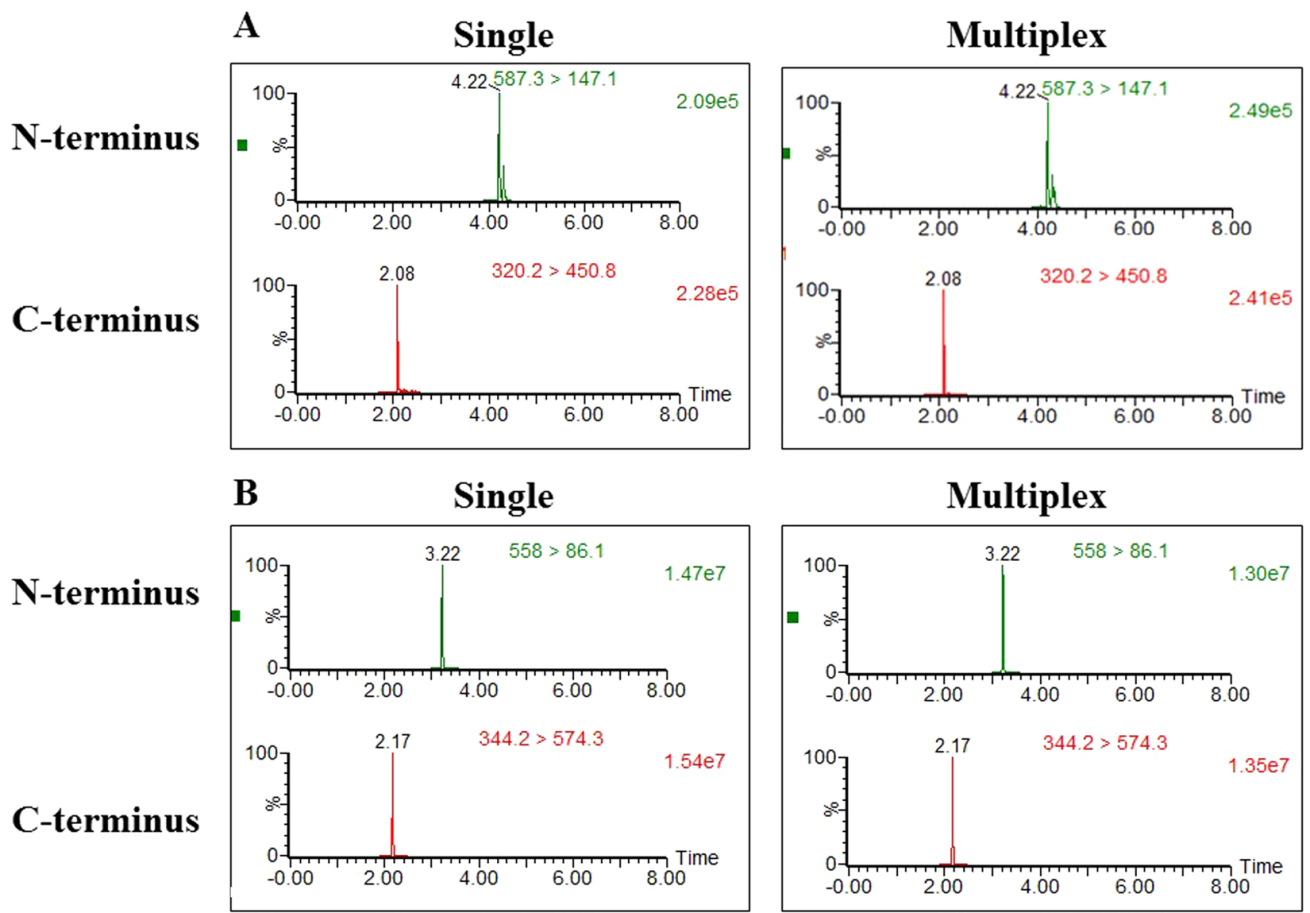
LC-MS-MS/MRM peak intensities for N- and C-terminal cleavage products in single versus multiplex BoNT/B (**A**) and BoNT/E (**B**) Endopep-MS assays. Upper panel: N-terminal cleavage product obtained in serum (0.5 MsLD_50_/ml) from single (left) and multiplex (right) Endopep-MS assays. Lower panel: C-terminal cleavage product obtained in serum (0.5 MsLD_50_/ml) from single (left) and multiplex (right) Endopep-MS assays.

As previously observed for BoNT/B and E, their activities were not reduced by the presence of other peptide substrates in the mixtures (Fig. 6).

**Figure 7:**
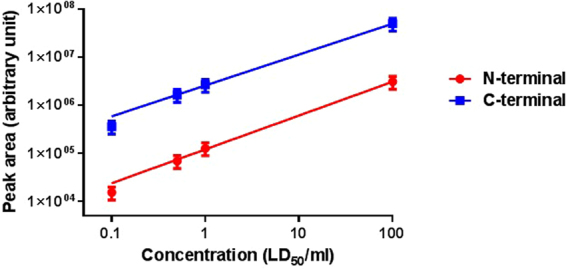
Linearity of the assay with the newly designed BoNT/A substrate. BoNT/A (0.1–100 LD_50_/ml) was analysed using the shortened peptide as a substrate. Cleavage products were analysed by LC-MS/MS. Data are presented on a logarithmic scale. Each experiment was repeated 3–4 times. The R^2^ values of the N- and C-terminal products were 0.99 and 0.98, respectively (P < 0.001 and P = 0.001 for the N and C-terminal fragment, respectively).

To test whether the assay performance was affected by the integration of the newly designed substrate, BoNT/A activity was tested using 0.1, 0.5, 1 and 100 MsLD_50_/ml preparations. As shown in Fig. 7, a linear curve was obtained for both cleavage products. The R^2^ values of the N- and C-terminal products were 0.99 and 0.98, respectively (P < 0.001 and P = 0.001 for the N and C-terminal fragment, respectively). A similar linearity was reported for the lower concentration range (0.1–1 MsLD_50_/ml) by Wang *et al*. using the longer optimized peptide. These results confirm that the assay performance was maintained using our newly designed peptide. In addition, the lowest concentration tested with the newly designed BoNT/A substrate, 0.1 MsLD_50_/ml, is the same as reported by Wang *et al*., indicating that the sensitivity of the assay was maintained as well.

**Figure 8:**
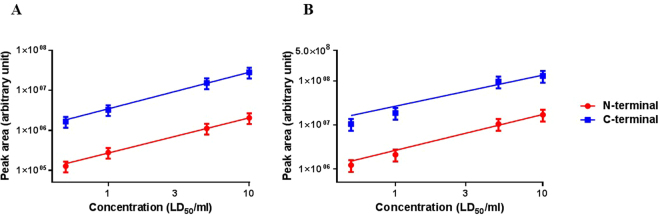
Linearity of the assay for BoNT/B and BoNT/E. 0.5–10 MsLD_50_/ml of BoNT/B (**A**) or BoNT/E (**B**) was analysed. Cleavage products were analysed by LC-MS/MS. Data are presented on a logarithmic scale. The experiments were repeated 2–3 times. The R^2^ values of the N- and C-terminal products were 0.99 and 0.99 for BoNT/B and 0.97 and 0.99 for BoNT/E, respectively (P < 0.001 and P = 0.001 for the N and C-terminal fragment of BoNT/B and P = 0.03 and P = 0.007 for the N and C-terminal fragment of BoNT/E, respectively).

Figure 8A and B shows the linearity of the multiplex assay for BoNT/E and BoNT/B, respectively, in a 0.5–10 MsLD_50_ concentration range.

The assay precision, <30%, was calculated for each toxin from 12 experiments (4 experiments for each toxin) using different serum samples (spiked with 0.5 MsLD50/ml toxin). The assay sensitivity for BoNT/B and BoNT/E in the multiplex Endopep-MS format was in the same order of magnitude as reported for the single format.

## Conclusions

We described herein the development of a multiplex Endopep-MS assay for the simultaneous detection of BoNT/A, B and E, which are the most relevant serotypes of human botulism. Diagnosis of BoNTs in the serum is very challenging, mostly because the levels of BoNTs in these clinical samples are expected to be very low and the amount of blood that can be drawn safely is extremely limited, particularly in infants. Therefore, the ability to multiplex the assay would be valuable. Multiplex assays have the advantages of reducing sample volumes and assay time without compromising performance. However, multiplexing raises the challenge of developing several assays that can be performed together with the same protocol, reagents and sample dilutions.

The Endopep-MS is an *in-vitro* activity assay that can be used to detect botulinum toxins^10,15,16^; this assay only requires a few hours to complete, is highly specific and is more sensitive than the gold standard *in-vivo* mouse bioassay^10,15,16^. The superior sensitivity, specificity and speed of the Endopep-MS assay have the potential to facilitate both diagnosis and treatment. However, until now, the Endopep-MS assay could not be performed concurrently for all BoNTs relevant to humans because the BoNT/E substrate interferes with BoNT/A activity.

In this study, we identified the molecular basis for this interference by studying the 3D-interface of both BoNT/A and BoNT/E substrates with the BoNT/A catalytic domain and showed how the overlap between the substrates reduced the binding of the BoNT/A substrate to the catalytic domain. Removing the overlapping fragment allowed the establishment of a sensitive multiplex Endopep-MS assay for BoNT/A, B and E. The use of this developed multiplex assay will reduce the serum sample volume and the time required. The multiplex assay should be a primary component in the human botulinum diagnostic toolkit based on its ability to provide clinically useful data with small amounts of sample in a clinically relevant timeframe.

## Material and Methods

### Ethics Statement

All animal experiments were performed in accordance with the Israeli Law and were approved by the Ethics Committee for animal experiments at the Israel Institute for Biological Research (protocol # M-72-2014). All efforts were made to minimize suffering.

#### Peptides

All peptides were synthesized by Proimmune, UK. The peptide substrate sequences were as follows: for BoNT/A - amino acids 189–204 of the SNAP-25 protein ^189^RTRIDEGNQRATR(Nle)LG^204^; for BoNT/B - amino acids 60–87 of the VAMP protein ^60^LSELDDRADALQAGASQFETSAAKLKRK^87^; and for BoNT/E - amino acids 167–186 of the SNAP-25 protein ^167^(Nle)GNEIDTQNRQIDRI(Nle)EKAD^186^. The lyophilized peptides were dissolved in water, and their concentrations were determined by UPLC using a peptide standard mixture (Sigma-Aldrich).

#### Toxins and Antibodies

*Clostridium botulinum* A, B, and E strains were obtained from the IIBR collection (A198, B592, and E450 respectively). Sequence analysis revealed compliance of the neurotoxin genes with serotypes 62 A (GenBank Accession Number M30196), Danish (GenBank Accession Number M81186) and NCTC11219 (GenBank Accession Number × 62683) of *C. botulinum* types A1, B1, and E respectively^17,18^.

Toxin complexes were prepared from concentrated supernatants from cultures grown for 6 days in anaerobic culture tubes. Toxins A and B had specific activities of 7.4 × 10^6^ and 1.5 × 10^7^ MsLD_50_/mg, respectively. Each toxin stock was incubated in 50 mM citrate buffer (pH = 5.5) at −70 °C. Toxins were further diluted to a working stock concentration in gelatine buffer (0.2% w/v gelatine in phosphate buffer, pH = 6.4). For determination of the lethal dose (mouse LD test), various doses of the toxin were injected intraperitoneally into groups of mice (n = 5) as described by Malizio *et al*.^19^, and lethality was calculated according to Spearman-Karber method^20^.

Rabbit anti-HC/A, anti-HC/B and anti-HC/E polyclonal antibodies were purified from the sera of hyperimmune rabbits that were immunized with the relevant HC as previously described^21^.

#### Endo-peptidase activity assay

Biotinylated polyclonal antibodies specific for the heavy chain of BoNT/A, B or E serotypes were bound to streptavidin magnetic beads (M-280 Dynabeads, Invitrogen). The beads were blocked with 5% BSA. Biotinylated antibodies were added to streptavidin magnetic beads at a 1:20 ratio (1 µl antibodies to 20 µl beads) and were incubated while shaking for 30 minutes at room temperature. The conjugated beads were washed three times with PBS.

Toxins (as indicated, 1 ml) were incubated while shaking (TalBoys, Troemner, NJ, USA) with 40 µl conjugated magnetic beads for 1 hour, 37 °C, 800 rpm. Next, the supernatant was removed and the beads were washed 3 times with decreasing volumes (1, 0.5 and 0.2 ml) of PBS containing 0.1% BSA and 0.025% tween, followed by 3 additional washing steps with 0.2 ml PBS (after the second wash with PBS, the beads were transferred to a new tube). After the final washing step, the supernatant was removed and the endo-peptidase reaction was performed.

The endo-peptidase activity assay was carried out as described previously^7,9,16^. Briefly, the reaction was performed in a 20 µl volume containing toxin-bound beads, 50 or 100 µM peptide substrate, 1 mM ZnCl_2_, 1% triton, 10 mM dithiothreitol (DTT), and 50 mM HEPES buffer (pH 7.3) at 37 °C with shaking at 800 rpm for 5 hours. The reaction was stopped by adding 2 µl of 99% formic acid, followed by centrifugation and addition of 180 µl of water. Beads were removed prior to MS analysis.

For multiplex assays, each toxin was incubated while shaking with a combination of BoNT/A, B and E-conjugated magnetic beads for 1 hour at 37 °C. The subsequent endo-peptidase activity step was carried out with peptide substrates for BoNT/A, B and E in a single tube.

Statistical analysis was conducted with 12 replicates (4 for each toxin), using different individual serum samples spiked with 0.5 MsLD_50_/ml. The precision obtained was better than 30%. For each serum sample, a control experiment, containing all the assay components except the toxin, was carried out.

The specificity of the assay was confirmed by showing that for any individual toxin tested, only cleavage products of the homologous peptide substrate were formed whereas no signal associated with the formation of cleavage products of the two other heterologous toxins was detected. This was confirmed for both BoNT/A, BoNT/B and BoNT/E.

#### In silico modelling

In silico modelling of SNAP-25 within the BoNT/E catalytic domain was based on the structure of SNAP-25 with the BoNT/A catalytic domain^12^ and the known 3D-structure of the BoNT/E catalytic domain (accession number 1T3A). In addition, all published interactions between SNAP-25 and BoNT/E catalytic domain were used for side chain-side chain constraints^13^. The calculations were performed using Pymol.

#### LC-MS/MS analysis

This analysis was performed on an Aquity UPLC-I-Class (SM-FTN) coupled to a Xevo TQ-S Triple Quadrupole mass spectrometer (Waters) equipped with an electrospray ionization (ESI) source in multiple reaction monitoring (MRM) mode. The cleavage products were chromatographically separated on a 150 × 2.1 mm i.d., 1.7 μm, 130 Å CSH-C18 column (Waters). The mobile phase consisted of 1% formic acid in H_2_O (eluent A) and 1% formic acid in acetonitrile:H_2_O (8:2 v/v; eluent B) in a linear gradient. Identification criteria included retention time and at least two MRM transitions for each cleavage product with the expected transition intensity ratios. The cleaved N- and C-terminal products were qualitatively evaluated in comparison to positive and negative control samples (normal human serum, NHS, with or without spiked toxin).

### Data availability

All data generated or analysed during this study are included in this published article.
